# CA-125 KELIM as an Alternative Predictive Tool to Identify Which Patients Can Benefit from PARPi in High-Grade Serous Advanced Ovarian Cancer: A Retrospective Pilot Diagnostic Accuracy Study

**DOI:** 10.3390/ijms25105230

**Published:** 2024-05-11

**Authors:** Dimitrios Zouzoulas, Dimitrios Tsolakidis, Panagiotis Tzitzis, Kimon Chatzistamatiou, Vasilis Theodoulidis, Iliana Sofianou, Grigoris Grimbizis, Eleni Timotheadou

**Affiliations:** 1 1st Department of Obstetrics & Gynecology, “Papageorgiou” Hospital, Aristotle University of Thessaloniki, 56403 Thessaloniki, Greece; 2 Department of Oncology, “Papageorgiou” Hospital, Aristotle University of Thessaloniki, 56403 Thessaloniki, Greece

**Keywords:** KELIM score, homologous recombination deficiency (HRD), Poly (ADP-ribose) polymerase inhibitors (PARPi)

## Abstract

BRCA mutation and homologous recombination deficiency (HRD) are the criteria for the administration of PARP inhibitor (PARPi) maintenance therapy. It is known that PARPi efficacy is related to platinum sensitivity and that the latter can be demonstrated from the CA-125 elimination rate constant (KELIM). This study aims to investigate if KELIM can be another tool in the identification of patients that could be benefit from PARPi therapy. Retrospective analysis of patients with high-grade serous advanced ovarian cancer that underwent cytoreduction and was further tested for HRD status. The HRD status was tested either by myChoice HRD CDx assay or by RediScore assay. KELIM score was measured in both neoadjuvant and adjuvant settings with the online tool biomarker-kinetics.org. A total of 39 patients had available data for estimating both HRD status and KELIM score. When assuming KELIM as a binary index test with the value 1 as the cut-off point, the sensitivity was 0.86, 95% CI (0.64–0.97) and the specificity was 0.83, 95% CI (0.59–0.96). On the other hand, when assuming KELIM as a continuous index test, the area under the curve (AUC) was 81% and the optimal threshold, using the Youden index, was identified as 1.03 with a sensitivity of 85.7% and a specificity of 83.3%. KELIM score seems to be a new, cheaper, and faster tool to identify patients that can benefit from PARPi maintenance therapy.

## 1. Introduction

The standard treatment protocol for patients facing advanced ovarian cancer typically involves debulking surgery and platinum-based chemotherapy, administered in varying sequences, followed by a phase of maintenance therapy. Despite the acknowledged significance of residual disease following cytoreduction as a crucial prognostic factor [1,2], the absence of a dependable indicator for tumor chemosensitivity poses a challenge in assessing the efficacy of first-line treatments [3]. Compounding this challenge is the fact that nearly 75% of ovarian cancer patients are diagnosed with an advanced stage of the disease, often challenging to accurately evaluate using conventional imaging methods [4]. Even with the application of the Response Evaluation Criteria in Solid Tumors (RECIST) [5], limitations persist. Consequently, there is a pressing need for precise outcome predictors to inform treatment decisions in the first-line setting, a concern recognized by both the European Society of Gynecological Oncology (ESGO) and the European Society of Medical Oncology (ESMO) [6].

The monitoring of CA-125 decline during chemotherapy has emerged as a valuable tool for predicting treatment response [7], particularly in circumventing the limitations of imaging techniques. This has been extensively studied in ovarian cancer patients [8,9]. Ongoing research in ovarian cancer patients has explored parameters such as the CA-125 nadir level, half-life value, and time to nadir as potential indicators [10]. Additionally, the Gynecologic Cancer InterGroup (GCIG) has defined CA125-based response as a 50% reduction in CA-125 levels sustained for at least 28 days [5]. However, neither approach has conclusively proven to accurately predict chemosensitivity, and their predictive value has come under scrutiny [11]. As the quest for more reliable indicators persists, refining these methods remains crucial for enhancing the precision of treatment strategies.

Recently, the ELIMination rate constant K (KELIM), a modeled kinetic parameter derived from CA-125 measurements over the initial 100 days of systemic therapy with at least three consecutive values of CA-125 at specific days during the cycles of systemic therapy, whether adjuvant or neoadjuvant chemotherapy, has demonstrated considerable promise as a valuable predictive tool [12]. This innovative approach involves a mathematical modeling method that does not rely on the absolute values of the biomarker but instead focuses on the longitudinal kinetics, specifically CA-125 elimination, throughout the course of treatment [13]. Remarkably, this method is independent of renal function. Numerous studies have substantiated its credibility as a reliable and independent prognostic factor, particularly in the first-line setting for assessing chemosensitivity [14,15,16,17]. KELIM’s association with radiological response during neoadjuvant chemotherapy, the likelihood of achieving complete cytoreduction during interval debulking surgery, the probability of platinum-resistant relapse, and its impact on patient progression-free survival (PFS) and overall survival (OS) further strengthens its clinical relevance. It is noteworthy that a higher KELIM corresponds to a more rapid CA-125 elimination, indicative of heightened chemosensitivity and a more favorable prognosis [18]. This provides clinicians with a valuable tool for predicting treatment outcomes and tailoring therapeutic strategies based on the individual dynamics of CA-125 elimination during the critical early stages of systemic therapy.

On the other hand, the landscape of maintenance therapy for ovarian cancer has undergone recent transformations with the introduction of Poly (ADP-ribose) polymerase inhibitors (PARPi). However, the question of which patients stand to derive genuine benefits from PARPi maintenance therapy remains a subject of contention. The well-established correlation between PARPi efficacy and platinum sensitivity in both the first-line and recurrent settings has added complexity to this discussion [19,20]. While PARPi activity is undeniably linked to BRCA mutations and homologous recombination deficiency (HRD) [21,22,23,24], the complete efficacy of these drugs remains not entirely explained by these two biomarkers, leaving substantial knowledge gaps. The conflicting results in the outcomes of ovarian cancer patients with germline BRCA1/2 mutations and the fact that some studies showed better survival in wild-type patients [25,26], while some BRCA1 patients were resistant to platinum-based chemotherapy agents [27], showed that not all ovarian cancer cells with BRCA1/2 mutation exhibit homologous recombination deficiency, thus further investigation was needed. Genomic instability is an evolving marker, that aims to solve this discrepancy. The hypothesis is that genomic instability can be attributed to defects in the HR pathway that maintains genomic stability [13]. There are two forms of genomic that can be considered as reflections of HR deficiency: the chromosomal alteration and the mutator phenotype, which can be quantified by the frequency of copy-number change (CNC) and the frequency of somatic mutation, respectively [28]. Additionally, HRD plays a crucial role in DNA double-strand break repair defects and serves as a highly predictive marker for primary platinum sensitivity, as tumor cells become incapable of repairing double-strand breaks induced by platinum [29,30]. Consequently, exploring a potential relationship between PARPi efficacy and KELIM, a robust predictor of chemosensitivity, becomes an intriguing avenue of investigation. 

This study’s primary objective is to conduct a comparative analysis of the KELIM score and HRD status as a potential alternative tool compared to HRD testing in identifying patients who could potentially benefit from PARPi maintenance therapy. By delving into this comparison, the aim is to shed light on novel avenues for patient stratification and personalized treatment approaches in the realm of ovarian cancer maintenance therapy.

## 2. Results

This retrospective pilot diagnostic accuracy cross-sectional study included 100 women, who were treated during the period of the study for histologically proven high-grade ovarian cancer in the Gynecological—Oncology Unit, 1st Department of Obstetrics & Gynecology, Aristotle University of Thessaloniki, “Papageorgiou” Hospital. After screening the patients’ records based on the inclusion and exclusion criteria, 39 patients were eligible for further analysis in this study.

Patients’ data are outlined in Table 1. A total of 39 patients had all the necessary data for both HRD status and CA-125 values, at the correct time frame in relation to cycles of chemotherapy during the first 100 days of treatment, in order to calculate the KELIM score through the online tool. The interplay between KELIM score and HRD status revealed notable findings, with approximately half (54%) of the patients exhibiting a favorable KELIM score (≥1). Similarly, an equivalent proportion (54%) demonstrated a positive HRD status. The mean age of the patients was 60 years old with a standard deviation of 10. Furthermore, concerning the timing of systemic treatment, our patients were equally distributed between the two groups: 22 (56%) women were offered neoadjuvant, while 17 (44%) adjuvant chemotherapy. The chemotherapeutic agents that were used in all patients were Paclitaxel and Carboplatin. All HRD-positive patients (54%) received PARPi maintenance therapy. Importantly, all patients underwent debulking surgery, with a significant majority (84%) achieving either complete or optimal cytoreduction, while 6 (16%) patients were sub-optimally debulked with a residual disease ≥1 cm.

**Figure 1:**
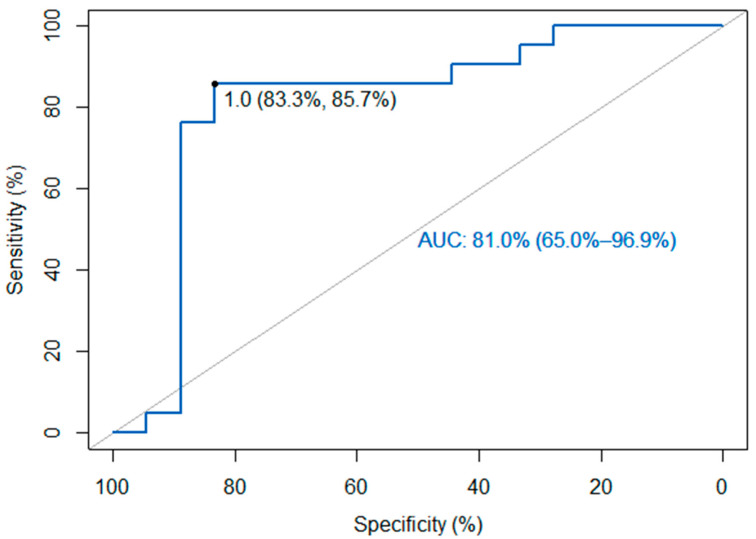
KELIM Roc curve compared to HRD.

The main endpoint of our study was to compare KELIM to HRD as a possible alternative tool to identify patients who could benefit from PARPi maintenance therapy. Our reference standard was the HRD test (either myChoice HRD CDx assay or by RediScore assay, which are both validated) and our index test was the KELIM score, through the online tool biomarker-kinetics.org/CA-125-neo or biomarker-kinetics.org/CA-125, based on the neoadjuvant or adjuvant setting. Firstly, when assuming the KELIM score as a binary index test with the value 1, as the cut-off point, which is also proposed by the creators of the model, the sensitivity was 0.86, 95% CI (0.64–0.97), and the specificity 0.83, 95% CI (0.59–0.96). The relevant data are presented in Table 2. On the other hand, when assuming KELIM as a continuous index test and constructing an ROC curve, the area under the curve AUC was 81% and the optimal threshold, using the Youden index, was identified as 1.03, which is in accordance with the proposal of the creators of KELIM, with a sensitivity of 85.7% and a specificity of 83.3%. Figure 1 shows the aforementioned data.

Furthermore, we aimed to see the correlation of the KELIM score with the Genomic Instability Score (GIS), from which the HRD status is calculated. Both values were tested as continuous variables to examine the hypothesis that as the GIS score elevates, so does the KELIM score. The Pearson correlation coefficient was used to investigate the linear correlation between the two sets of data. The results revealed a strong correlation between the KELIM score and GIS in the same direction, which was statistically significant (r = 0.51 [95% CI: 0.23–0.71], *n* = 39, *p* < 0.001), meaning that as the value of one variable goes up, the value of the other also tends to do so. 

**Figure 2:**
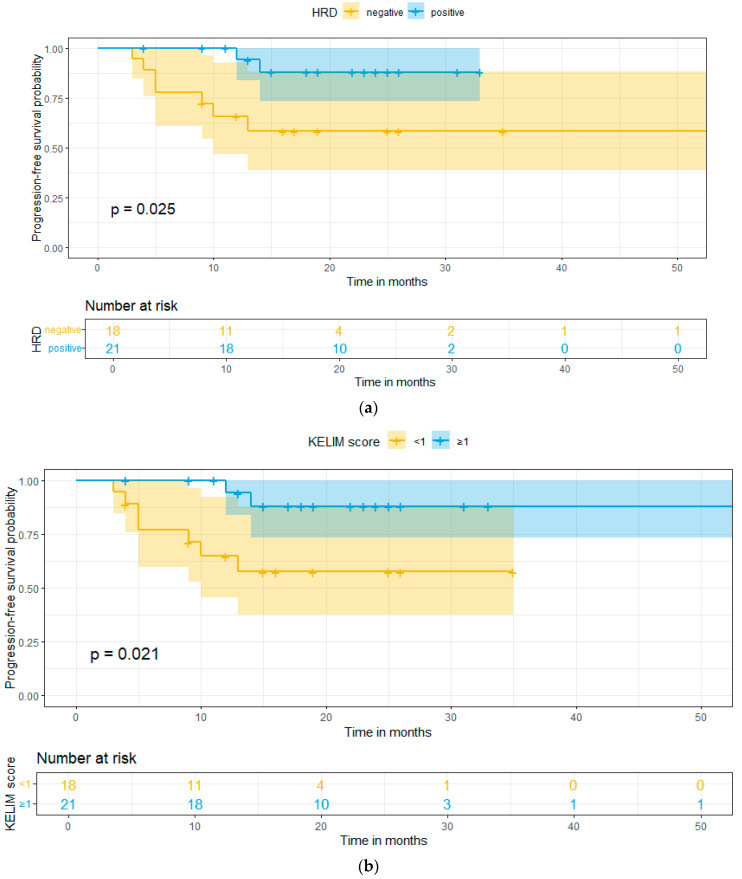
(**a**) Progression free survival based on HRD status. (**b**) Progression free survival based on KELIM score.

**Figure 3:**
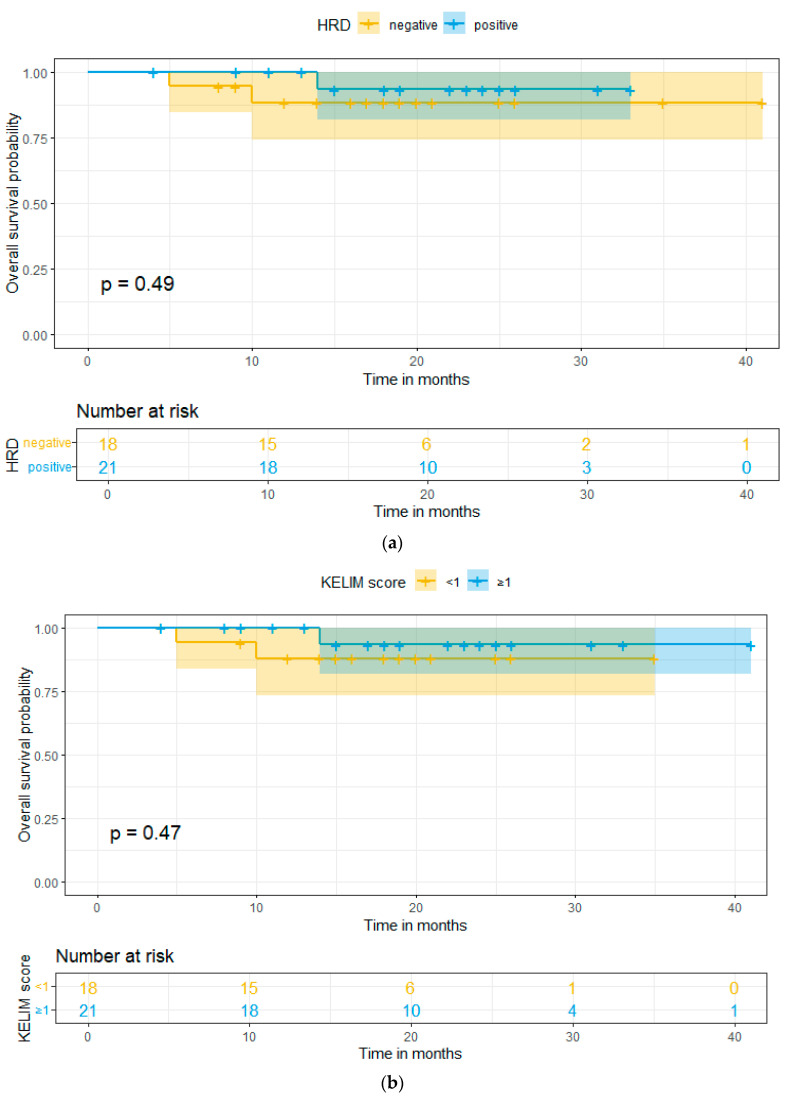
(**a**) Overall survival based on HRD status. (**b**) Overall survival based on KELIM score.

Last but not least, concerning survival data, the patients of this study had a mean follow-up of 19 months, and the progression-free survival (PFS) and overall survival (OS) were calculated, for both KELIM score and HRD status. There was a statistically significant difference in the PFS between HRD-positive and -negative patients (*p*-value = 0.025) and also in the PFS between KELIM-favorable and -unfavorable patients (*p*-value = 0.021). In addition, a better PFS was observed in the HRD-positive patients, with the 2-year PFS at 87.5% and 60% for HRD-positive and -negative patients, respectively. The same results were verified in the KELIM-favorable patients, with the 2-year PFS at 87.5% and at 60% for KELIM-favorable and -unfavorable patients, respectively. The above-mentioned results are presented in Figure 2a,b. On the one hand, concerning OS, no statistical significance was found between HRD-positive and -negative patients (*p*-value = 0.49) and also in KELIM-favorable and -unfavorable patients (*p*-value = 0.47). The aforementioned results are presented in Figure 3a,b.

## 3. Discussion

The primary objective of our study was to investigate the potential utility of the KELIM score as an additional tool compared to HRD testing for identifying patients who may benefit from PARPi therapy. To achieve this, we conducted a comparative analysis with HRD status, currently regarded as the gold standard test, along with BRCA mutation, for the administration of maintenance PARPi therapy. Furthermore, we also investigated the association of KELIM score and GIS as continuous variables. We designed a retrospective diagnostic accuracy cross-sectional study, structured according to the PICO model: The population was high-grade serous advanced ovarian cancer patients, the index test was KELIM score, the control was HRD test and outcomes were the sensitivity–specificity and the AUC of the new diagnostic test.

A total of 39 patients were included in the study and they almost equally underwent neoadjuvant (56%) and adjuvant (44%) chemotherapy, with the same base chemotherapy agents (Paclitaxel + Carboplatin). PARPi maintenance therapy was offered to all HRD-positive patients (54%). Concerning surgery, the majority of the patients (84%) underwent a complete or optimal debulking surgery, while a small percentage (16%) had a residual disease of ≥ 1 cm (suboptimal debulking). Furthermore, almost half (54%) of the patients had a positive HRD status and a favorable (≥ 1) KELIM score. When assuming the KELIM score as a binary index test with a cut-off value of 1 the sensitivity was 86% and the specificity 83%, results that classify the diagnostic accuracy of the KELIM score as very good. On the other hand, when assuming the KELIM score is a continuous index test and constructing an ROC curve, the AUC was 81%, which is considered an excellent score. The optimal threshold value of the diagnostic test was calculated at 1.03, with a sensitivity of 85.7% and a specificity of 83.3%. Moreover, when assuming the KELIM score to be a continuous variable, we investigated its linear association with the GIS, which is also a continuous variable. We used the Pearson correlation coefficient and found a strong association between the variables (r = 0.51 [95% CI: 0.23–0.71], *n* = 39, *p* < 0.001) because results > 0.5 are considered a high degree of correlation. These promising results, when comparing the KELIM score with both HRD status and GIS, show that KELIM is at least as good as the test used at this point to choose the patients that will be administered PARPi maintenance therapy.

The mean follow-up period of the patients was 19 months and the progression-free and overall survival were calculated. The survival analysis demonstrated a statistically significantly better PFS in HRD-positive and KELIM-favorable patients, with a higher 2-year PFS for both categories, when compared to HRD-negative and KELIM-unfavorable patients, respectively. However, no statistical significance was observed in either analysis with HRD status or KELIM score. In addition, no statistical significance was observed in the OS in either HRD status or KELIM score. This could be attributed to the small follow-up period and the relatively small sample size, that led to a low event occurrence (only three deaths were reported in the whole cohort of patients).

To the best of our knowledge, this is the only diagnostic accuracy study in the literature considering the KELIM score as an alternative tool to HRD status for the prediction of PARPi response. Two recent meta-analyses [7,18] showed that KELIM is an independent prognostic biomarker for survival outcomes and that can predict chemosensitivity. The first meta-analysis [7] aimed to investigate the prognostic value of the KELIM score for progression-free and overall survival in primary and recurrent settings. The study included 14.444 patients with epithelial ovarian cancer and showed that a favorable KELIM score is associated with a significantly better PF and OS. The second meta-analysis [18] included 5.884 patients and assessed the prognostic and surrogate values of the KELIM score with respect to those of surgery outcomes in the primary setting. Three prognostic groups were identified (good prognosis: favorable KELIM and optimal surgery, intermediate prognosis: unfavorable KELIM or suboptimal surgery, poor prognosis: unfavorable KELIM and suboptimal surgery) and the study confirmed the strong prognostic values of the KELIM score for survival, independently from surgery completeness.

There are only a few studies in the literature that investigate the role of KELIM as a potential indicator of which patients might benefit from PARPi maintenance therapy [31,32,33]. The most relevant one is a post hoc study [31], which used the data of 854 patients that were enrolled in the VELIA trial and explored the prognostic value of KELIM with the benefit from veliparib. The study included patients that underwent both adjuvant and neoadjuvant chemotherapy but had, as a “control” test, both HRD and BRCA status. The results agree with our findings and suggest that the KELIM score might be another complementary determinant of PARPi efficacy. Moreover, a retrospective study from Canada [32], that was published only as an abstract from the ASCO 2022 meeting, included 70 patients and stated that KELIM can be used to aid clinical decision-making when HRD testing is unfunded. Another post hoc study [33], that used the datasets of ARIEL2 and STUDY 10, concluded that KELIM is a pragmatic indicator of rucaparib efficacy, but in the recurrent setting and not in the first-line setting.

Last but not least, the KELIM score has been evaluated in the neoadjuvant setting as a predictive tool for PFS, OS, and also for complete interval debulking surgery. Many studies [15,17,34,35,36] confirmed the above-mentioned results and a recent publication [37] from the same group of authors showed that the KELIM score is an independent predictive tool of surgery completeness in the neoadjuvant setting. Moreover, a recent narrative review of the literature [3], from the same authors that created the KELIM score, in the chapter about the potential utilities of KELIM, states that it may be a useful tool for identifying patients who might benefit from PARPi maintenance therapy. However, they underline that further studies are needed to better clarify this possible association between KELIM and PARPi efficacy, which is exactly the knowledge gap that our study tries to fill.

This is the first diagnostic accuracy study that investigates the role of KELIM score, compared to HRD status, in identifying patients that could benefit from PARPi maintenance therapy. All the required parameters were collected from an online system, therefore minimizing the percentage of missing important data. It is of high importance to state that the dataset, on which this study was conducted, comes from an ESGO-certified center for Advanced Ovarian Cancer Surgery for over a decade, thus ensuring the high quality of the data. Nevertheless, the main limitation of our study is the relatively small sample size that was included in the final analysis due to its pilot design. Moreover, the follow-up period for the survival analysis was certainly not long enough to extract safe results about overall survival, and the promising results about progression-free survival should be taken into consideration with caution. Another possible limitation of our study is its retrospective nature.

The results of our study could have a huge impact on everyday clinical practice because the KELIM score seems to be as good as HRD status for aiding in the selection of patients that will benefit from PARPi maintenance therapy while being an easy, cheap, and reproducible tool to use in clinical practice. The value of the KELIM score seems to be an alternative triage tool in world areas where HRD testing is unavailable. Further prospective studies are imperative to validate these promising results before the KELIM score is established as a diagnostic test for PARPi efficacy.

## 4. Materials and Methods

### 4.1. Study Characteristics

In conducting a comprehensive retrospective analysis, we meticulously examined the sequential medical records of all female patients diagnosed with ovarian cancer and treated within the 1st Department of Obstetrics & Gynecology at AUTh, “Papageorgiou” General Hospital. The retrospective review encompassed a timeframe extending from 1 January 2019 to 31 December 2022. Specifically, our focus was on identifying individuals who underwent primary or interval debulking surgery within this specified duration. Remarkably, a cohort of 100 patients received a diagnosis of ovarian cancer during the aforementioned period. In adherence to ethical protocols, we sought and obtained written approval from the Institutional Review Board of the hospital, ensuring that our retrospective analysis aligns with the highest standards of research integrity and patient confidentiality.

### 4.2. Patients

Inclusion criteria:

- Histological confirmation of ovarian cancer.
- Surgical treatment at our Gynecological–Oncology Unit.

Exclusion criteria:

- Any other histological type except high-grade serous.
- Patients not tested for HRD.
- Missing important registry data of CA-125 values to calculate KELIM.

**Figure 4:**
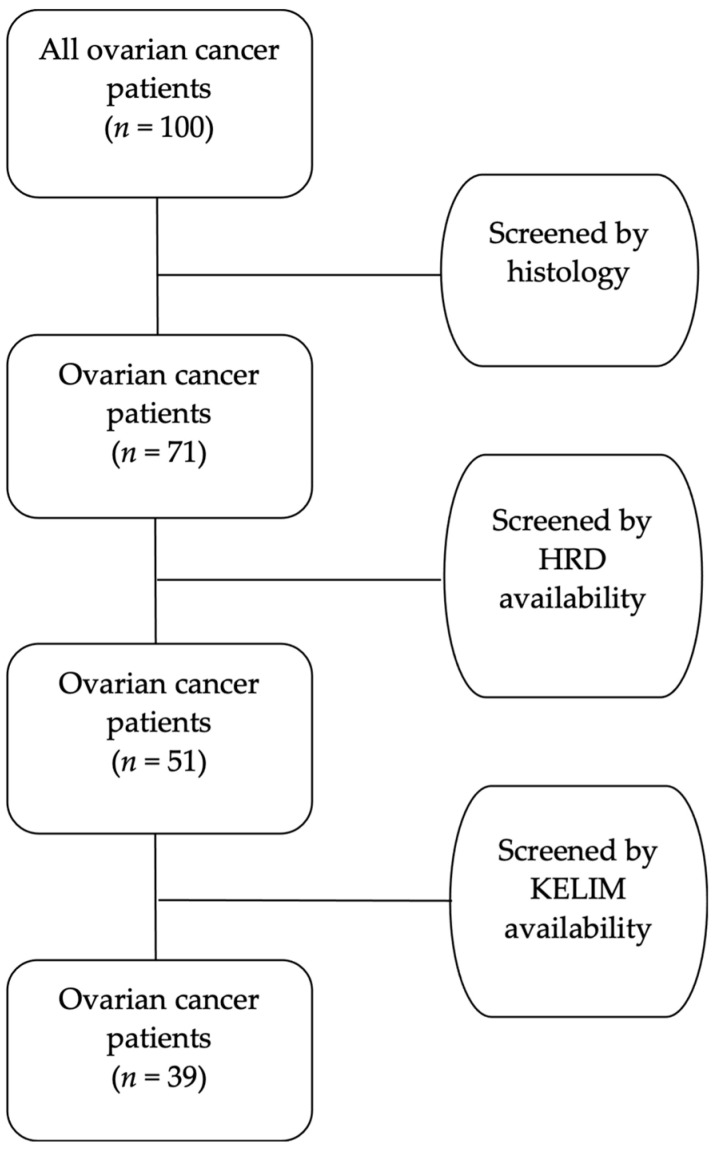
Patients’ selection flowchart.

The application of the aforementioned stringent criteria resulted in the exclusion of 29 out of the initial 100 women diagnosed with ovarian cancer, primarily due to histological types other than high-grade serous. Additionally, 20 women were omitted from the study as they did not undergo testing for homologous recombination deficiency (HRD). Another 12 women were excluded due to the absence of crucial registry data related to CA-125 values, which are essential for calculating KELIM. Consequently, after thorough screening and exclusion based on these criteria, a refined cohort of 39 women diagnosed with high-grade serous ovarian cancer emerged as eligible for further analysis. Because this study was designed as a pilot diagnostic accuracy study, the sample of 39 patients was deemed enough to continue the statistical analysis. It is crucial to note that this final cohort exhibits a careful elimination of duplicate data and addresses the issue of missing values that could potentially impact the integrity of the analysis. The patient selection process (flowchart) is visually represented in Figure 4, providing a transparent overview of the refined cohort and the exclusion criteria applied to ensure the robustness of the subsequent analysis.

### 4.3. Data Collection

The data collection process spanned a period of one month, during which we scrupulously gathered information. To streamline and standardize the data collection process, we leveraged the online registry of our Gynecological–Oncology Unit containing all pertinent details from the patients’ medical records. In an effort to mitigate potential inconsistencies stemming from variations in data collection dates, a systematic approach was adopted. A uniform data collection sheet, formatted as an Excel file, was employed for the retrospective compilation of patients’ medical records. This standardized sheet served as a systematic tool to ensure consistency and accuracy in the recorded information. The data collection sheet encompassed a range of essential information, contributing to a holistic understanding of each patient’s medical history. This included the following information:

- Patient’s identifiers:
  ○ Name
  ○ Hospital identification number
- Patient’s age
- Tumor marker CA-125 serial values during chemotherapy
- KELIM Score
- Homologous Recombination Deficiency (HRD) status
- Genomic Instability Score (GIS)
- Residual Disease after debulking surgery
- Chemotherapy:
  ○ Adjuvant
  ○ Neoadjuvant
- Maintenance therapy (PARPi)
- Time-related data:
  ○ Date of surgery
  ○ Date of recurrence or disease progression
  ○ Date of last follow-up or death

The determination of HRD status was tested either by myChoice HRD CDx assay [38] or by RediScore assay [39]. Both assays assess the genomic instability score (GIS score) and the cut-off point for HRD-positive status was 42 or greater (≥42). This dual-testing approach ensures a robust evaluation of homologous recombination deficiency. On the other hand, the KELIM score was measured in both neoadjuvant and adjuvant settings with the online tool biomarker-kinetics.org/CA-125-neo or biomarker-kinetics.org/CA-125, respectively. The dates of every cycle of chemotherapy were entered and the relevant values of CA-125 within the first 100 days from the start of neoadjuvant or adjuvant chemotherapy. Preferably the CA-125 values before cycles 2, 3, and 4 were used to calculate the KELIM score, but if one was missing, the CA-125 value prior to the 1st cycle of chemotherapy (within 7 days from neoadjuvant chemotherapy start) was considered. The KELIM score was analyzed as a continuous variable and as a binary index test with a cut-off point of 1 or greater (≥1) for favorable results.

### 4.4. Statistical Analysis

In the statistical analysis, the baseline characteristics of the patients who participated in the study were calculated. There was no case of missing data. Continuous variables are demonstrated as means with standard deviation (SD) and categorical variables as frequencies and percentages *n* (%). A receiver operating characteristic (ROC) curve analysis and the Youden index (J) [40,41] of the KELIM score were performed to identify the most appropriate cut-off value for the ovarian cancer diagnosis. The sensitivity, specificity, positive predictive value, negative predictive value, and area under the curve (AUC) with 95% confidence intervals (CI) were calculated. The linear correlation between continuous variables was calculated with the Pearson correlation coefficient. Progression-free survival (PFS) and overall survival (OS) analyses were performed using the Kaplan–Meier curves and groups were compared using the log rank. Progression-free survival was defined as the time interval between the date of surgery and the date of first recurrence or disease progression, while overall survival was defined as the time interval from surgery to the date of death or last follow-up. A test of normality was conducted using the Shapiro–Wilk test. All reported *p*-values were two-tailed at a 5% significance level. We analyzed data using R statistical software (R Project for Statistical Computing), version 4.3.0 using the package called pROC [42].

## 5. Conclusions

The KELIM score stands as a novel, cost-effective, and easily reproducible biomarker, offering a streamlined approach to identifying patients poised to benefit from PARPi maintenance therapy, especially when compared to the conventional assessment of HRD/BRCA status. This emerging biomarker presents a promising avenue for refining patient stratification, enhancing treatment efficacy, and optimizing resource utilization in the context of PARPi maintenance therapy. Therefore, it is recommended that future investigations in the field of ovarian cancer therapy embrace the inclusion of the KELIM score as a fundamental component in their study designs. This will contribute to the ongoing evolution of precision medicine, fostering a deeper comprehension of the dynamics between KELIM scores and treatment outcomes in the era of PARPi.

## Figures and Tables

**Table 1:** Patient Characteristics.

		Number of Patients (n)	Percentage (%)
Age (years)		mean: 60	SD: 10
KELIM score			
	<1	18	46
	≥1	21	54
HRD status			
	Positive	21	54
	Negative	18	46
Chemotherapy			
	Neoadjuvant	22	56
	Adjuvant	17	44
Residual disease (cm)			
	0	22	56
	<1	11	28
	≥1	6	16

**Table 2:** KELIM Index test.

	Point of Estimates	95% CIs
Sensitivity	0.86	(0.64–0.97)
Specificity	0.83	(0.59–0.96)
Positive Predictive Value	0.86	(0.64–0.97)
Negative Predictive Value	0.83	(0.59–0.96)

## Data Availability

The data presented in this study are available on request from the corresponding author for the reproducibility of this study if such is requested, due to institution privacy reasons.
